# Prevalence of Urinary Stones and Their Associated Risk Factors in the Jazan Region of Saudi Arabia

**DOI:** 10.7759/cureus.77465

**Published:** 2025-01-15

**Authors:** Ahmed A Bahri, Ali M Shawish, Abdulrahman Y Safhi, Raed E Jarram, Ali A Zalah, Salem M Ayyashi, Ghadeer A Qumayri, Ahlam H Hakami, Ali E Abu Hayyah, Ebtehal M Hummdi, Ohoud A Alfaifi, Fahad A Alghamdi, Nada A Alghamdi

**Affiliations:** 1 College of Medicine, Jazan University, Jazan, SAU; 2 College of Nursing, Jazan University, Jazan, SAU; 3 College of Pharmacy, Jazan University, Jazan, SAU; 4 College of Medicine, Albaha University, Albaha, SAU; 5 College of Medicine, King Saud bin Abdulaziz University, Jeddah, SAU

**Keywords:** cross-sectional study, family history, hypertension, jazan, kidney stones, nephrolithiasis, prevalence, risk factors, saudi arabia, smoking

## Abstract

Background: Kidney stones, commonly calcium-based, form due to urine supersaturation and are influenced by factors such as pH, diet, and comorbidities. Symptoms include pain, hematuria, and infections. Studies indicate a higher prevalence in men and obese individuals. Risk factors include gender, climate, low fluid intake, and metabolic syndrome. Limited research has been conducted in Saudi Arabia. This study aimed to evaluate the prevalence and associated risk factors of kidney stones among participants in the Jazan Region, Saudi Arabia.

Methodology: This cross-sectional study included 386 participants from the Jazan region of Saudi Arabia. Data on kidney stone prevalence and risk factors were collected using structured questionnaires. Associations were analyzed using univariate and multivariate analyses, with statistical significance set at *p* < 0.05.

Results: The study assessed the prevalence of kidney stones and associated risk factors among 386 participants in Jazan, Saudi Arabia. More than half of the participants (n = 214, 55.4%) reported kidney stones, with male participants (n = 141, 65.9%) significantly more affected than female participants (n = 73, 42.4%; *p* < 0.001). Older age groups, particularly 51-60 years (n = 29, 78.4%) and >60 years (n = 5, 83.3%), showed higher prevalence (*p* < 0.001). Smoking (n = 87, 73.1%; *p* < 0.001), high blood pressure (n = 77, 81.9%; *p* < 0.001), and family history (n = 93, 77.5%; *p* < 0.001) were significant risk factors. The adjusted analysis identified smoking (odds ratio (OR) 1.776; *p* = 0.033), hypertension (OR 3.439; *p* = 0.001), and family history (OR 3.085; *p* < 0.001) as strong predictors. Higher education was protective (OR 0.563; *p* < 0.001), while BMI and other comorbidities showed no significant associations.

Conclusion: This study highlights a high prevalence of kidney stones in Jazan, Saudi Arabia, with significant predictors including male gender, older age, smoking, hypertension, and family history. Higher education was found to be protective. These findings underscore the importance of targeted prevention strategies addressing modifiable risk factors and promoting public health awareness.

## Introduction

Kidney stones, also referred to as renal calculi or nephrolithiasis, are a condition characterized by the formation of crystal deposits inside the kidney. Ideally, these deposits are expelled from the body painlessly through the urethra. However, larger calculi can cause severe pain and often require medical intervention. Approximately 80% of patients with renal calculi develop calcium-based stones, which are composed of either calcium phosphate or calcium oxalate. Other types of kidney stones include cystine, uric acid, and struvite stones [1].

Urine supersaturation triggers the precipitation of solutes in the urinary tract, leading to crystal formation and, subsequently, kidney stones [2]. Additionally, factors such as urine pH play a crucial role in the development of kidney crystals [2]. In the early stages of nephrolithiasis, patients may be asymptomatic. However, as the disease progresses, symptoms such as hydronephrosis, flank pain, hematuria, urinary tract infections, urine flow obstruction, and obstructive uropathy may manifest [1].

In the United States, a study involving 10,521 participants reported a kidney stone prevalence rate of 11% (n = 1,157) and a 12-month incidence rate of 2.1% (n = 221) [3]. Similarly, another US study evaluating kidney stone frequency among 12,110 participants found a prevalence of 8.8% (n = 1,066). The study also noted a higher occurrence of kidney stones among obese males compared to other groups [4].

A retrospective study conducted in Saudi Arabia’s Eastern Region analyzed the characteristics and types of kidney stones among 235 patients. Of these, 175 (74.5%) had renal calculi, with calcium oxalate being the most common type (n = 133, 76%) [5]. Environmental factors play a significant role in the pathophysiology of kidney stones, with risk factors varying among population subsets. Research has linked urinary stones to factors such as gender, ethnicity, geography, occupation, hot climates, and unhealthy diets high in caffeine, salt, dairy, animal proteins, and fat [6].

Additionally, individuals with a history of kidney stones have a 15% likelihood of recurrence within the first year and a 50% likelihood over the next 10 years [7, 8]. Studies indicate that high fluid intake significantly reduces the risk of kidney stones, while low fluid consumption is strongly associated with their occurrence and recurrence [9].

Kidney stone development has also been linked to comorbidities such as metabolic syndrome. Patients with three or more characteristics of metabolic syndrome are at a significantly higher risk of developing kidney stones [10]. Despite the clinical significance of kidney stones and their potential long-term complications, there is limited research on their prevalence and risk factors among the Saudi population. Therefore, this study aimed to investigate the incidence, prevalence, and associated risk factors of kidney stones in the population of Jazan, Saudi Arabia.

## Materials and methods

Study design

A cross-sectional design was employed to determine the prevalence and associated risk factors for urinary stones among the population of Jazan, Saudi Arabia. The study was conducted over three months, from October 2024 to December 2024.

Sample size calculation

The required sample size was calculated using the Raosoft sample size calculator (Raosoft Inc., Seattle, WA, USA; raosoft.com). To achieve a 95% confidence interval with a 5% margin of error, 377 participants were needed.

Sampling technique

A random sampling method was utilized to select participants, ensuring a representative sample of the population of Jazan. Data collection was conducted through an online questionnaire (Appendix A).

Inclusion and exclusion criteria

All residents of the Jazan region who agreed to participate in the study were included. However, individuals with cognitive impairments, those unable to respond to the questionnaire, and those who refused to participate were excluded from the study.

Statistical analysis

Data entry and statistical analyses were performed using IBM SPSS Statistics software, version 26 (IBM Corp., Armonk, NY, USA). Descriptive statistics were used to summarize demographic characteristics and other variables of interest, providing a clear overview of the study population. Inferential analyses were conducted to explore associations between variables. The chi-square test was employed to assess relationships between categorical variables, while Fisher’s exact test was applied when appropriate. Binary logistic regression was performed to identify adjusted predictors for renal stones. This approach allowed for a deeper understanding of the factors contributing to the formation of urinary stones while accounting for potential confounding variables. All statistical analyses were executed using IBM SPSS Statistics software, version 29 (IBM Corp.), to ensure accurate and reliable results.

## Results

A total of 386 participants from the Jazan region, Saudi Arabia, were included in this study to assess the prevalence of kidney stones and associated risk factors (Table 1). The sample comprised predominantly male participants (n=214, 55.4%) and Saudi nationals (n=317, 82.1%). Most participants were aged between 36-50 years (n=171, 44.3%) and held a diploma (n=132, 34.2%) or university-level education (n=104, 26.9%). Employment outside the health sector was common, with 187 participants (48.4%) reporting such roles. Regarding BMI, the majority of participants were classified as overweight (n=170, 44.0%). The prevalence of kidney stones was notably high, with more than half of the participants (n=214, 55.4%) reporting a history of stones. Smoking was reported by 119 participants (30.8%), while high blood pressure (n=94, 24.4%), diabetes (n=67, 17.4%), and a family history of kidney stones (n=120, 31.1%) were also common. Less prevalent factors included chronic kidney disease (n=19, 4.9%), hyperthyroidism (n=17, 4.4%), and vitamin D supplement usage (n=51, 13.2%).

**Table 1:** Sociodemographic parameters and the prevalence of kidney stones among the participants (n=386)

Parameter	Category	Frequency (n)	Percentage (%)
Sex	Female	172	44.6
	Male	214	55.4
Age	18–25 years	48	12.4
	26–35 years	124	32.1
	36–50 years	171	44.3
	51–60 years	37	9.6
	>60 years	6	1.6
Nationality	Non-Saudi	69	17.9
	Saudi	317	82.1
Educational level	Up to primary school	32	8.3
	Intermediate or secondary school	118	30.6
	Diploma	132	34.2
	University or higher	104	26.9
Occupation	Unemployed	131	33.9
	Work outside the health sector	187	48.4
	Work in the health sector	68	17.6
BMI	Underweight	32	8.3
	Normal weight	133	34.5
	Overweight	170	44.0
	Obese	51	13.2
Kidney stones	No	172	44.6
	Yes	214	55.4

Table 2 demonstrates the associations between sociodemographic factors and kidney stone prevalence. Significant gender differences were observed, with males (n=141, 65.9%) more frequently affected than females (n=73, 42.4%) (p < 0.001). Age was also a significant factor; participants aged 51-60 years (n=29, 78.4%) and those over 60 years (n=5, 83.3%) had notably higher prevalence rates (p < 0.001). Educational level correlated significantly with kidney stone prevalence, with individuals having up to primary school education exhibiting the highest prevalence (n=25, 78.1%; p = 0.002). Occupational differences were evident, as those employed outside the health sector had a higher prevalence (n=116, 62.0%) (p = 0.041). However, no significant associations were observed for nationality (p = 0.547) or BMI categories (p = 0.199).

**Table 2:** Association between sociodemographic parameters and the prevalence of kidney stones (univariate analysis)

Parameter	Category	No (n, %)	Yes (n, %)	p-value
Sex	Female	99 (57.6%)	73 (42.4%)	<0.001
	Male	73 (34.1%)	141 (65.9%)	
Age	18–25 years	31 (64.6%)	17 (35.4%)	<0.001
	26–35 years	51 (41.1%)	73 (58.9%)	
	36–50 years	81 (47.4%)	90 (52.6%)	
	51–60 years	8 (21.6%)	29 (78.4%)	
	>60 years	1 (16.7%)	5 (83.3%)	
Nationality	Non-Saudi	33 (47.8%)	36 (52.2%)	0.547
	Saudi	139 (43.8%)	178 (56.2%)	
Educational Level	Up to primary school	7 (21.9%)	25 (78.1%)	0.002
	Intermediate or secondary	52 (44.1%)	66 (55.9%)	
	Diploma	53 (40.2%)	79 (59.8%)	
	University or higher	60 (57.7%)	44 (42.3%)	
Occupation	Unemployed	67 (51.1%)	64 (48.9%)	0.041
	Work outside the health sector	71 (38.0%)	116 (62.0%)	
	Work in the health sector	34 (50.0%)	34 (50.0%)	
BMI	Underweight	16 (50.0%)	16 (50.0%)	0.199
	Normal weight	64 (48.1%)	69 (51.9%)	
	Overweight	76 (44.7%)	94 (55.3%)	
	Obese	16 (31.4%)	35 (68.6%)	

Table 3 outlines sociodemographic predictors of kidney stones among the participants. Being male significantly increased the likelihood of developing kidney stones, with male participants having 2.758 times the odds compared to female participants (95% CI: 1.763-4.314, p < 0.001). Age was a significant predictor, with each additional year increasing the odds by 49.3% (Exp(B) = 1.493, 95% CI: 1.145-1.946, p = 0.003). Higher educational attainment was protective, reducing the odds of kidney stones by 44% (Exp(B) = 0.563, 95% CI: 0.422-0.751, p < 0.001). Employment status also played a role, with employed individuals having 1.583 times the odds of developing kidney stones (95% CI: 1.089-2.302, p = 0.016). Nationality and BMI were not significant predictors (p-values of 0.661 and 0.367, respectively).

**Table 3:** Adjusted sociodemographic predictors of kidney stones (multivariate analysis)

Variable	B	Sig.	Exp(B)	95% CI
Gender (Male)	1.014	0.000	2.758	1.763, 4.314
Age	0.401	0.003	1.493	1.145, 1.946
Nationality (Saudi)	0.129	0.661	1.137	0.640, 2.022
Higher educational level	-0.574	0.000	0.563	0.422, 0.751
Occupation (Yes)	0.459	0.016	1.583	1.089, 2.302
BMI	0.126	0.367	1.135	0.862, 1.494
Constant	-0.555	0.363	0.574	

Table 4 presents the associations between risk factors and kidney stone prevalence using univariate analysis. Smoking was strongly associated, with 73.1% of smokers (n=87/119) having kidney stones compared to 47.6% of non-smokers (n=127/267), a statistically significant difference (p < 0.001). High blood pressure was another significant factor, with 81.9% of hypertensive individuals (n=77/94) reporting kidney stones, compared to 46.9% of those without hypertension (n=137/292) (p < 0.001). Diabetes also increased the risk, with 74.6% of diabetics (n=50/67) affected versus 51.4% of non-diabetics (n=164/319) (p < 0.001). Family history of kidney stones was a critical factor, with 77.5% of participants with a family history (n=93/120) reporting stones compared to 45.5% of those without (n=121/266) (p < 0.001). Other factors, such as inflammatory bowel disease, gout, chronic kidney disease, and thyroid disorders, as well as the use of vitamins, minerals, and supplements, showed no significant associations.

**Table 4:** Association between different risk parameters and the prevalence of kidney stones (univariate analysis)

Risk parameter		Presence of kidney stones (N%)	Sig. value
Smoking	No	140 (52.4%)	<0.001
	Yes	32 (26.9%)	
Inflammatory bowel disease	No	166 (45.1%)	0.326
	Yes	6 (33.3%)	
High blood pressure	No	155 (53.1%)	<0.001
	Yes	17 (18.1%)	
Diabetes	No	155 (48.6%)	<0.001
	Yes	17 (25.4%)	
Gout	No	165 (44.2%)	0.493
	Yes	7 (53.8%)	
Chronic kidney disease	No	167 (45.5%)	0.101
	Yes	5 (26.3%)	
Hyperthyroidism	No	167 (45.3%)	0.199
	Yes	5 (29.4%)	
Hypothyroidism	No	166 (44.3%)	0.549
	Yes	6 (54.5%)	
Hyperparathyroidism	No	168 (44.6%)	1.000
	Yes	4 (44.4%)	
Vitamin D pills	No	144 (43.0%)	0.111
	Yes	28 (54.9%)	
Calcium pills	No	154 (43.8%)	0.303
	Yes	18 (52.9%)	
Diuretics	No	169 (45.3%)	0.113
	Yes	3 (23.1%)	
Family history of kidney stones	No	145 (54.5%)	<0.001
	Yes	27 (22.5%)	

Table 5 highlights the adjusted predictors for kidney stones. Smoking remained a significant risk factor, with smokers having 1.776 times the odds of developing kidney stones compared to non-smokers (Exp(B) = 1.776, 95% CI: 1.047-3.015, p = 0.033). Hypertension was a strong predictor, with hypertensive individuals 3.439 times more likely to develop kidney stones (Exp(B) = 3.439, 95% CI: 1.663-7.110, p = 0.001). A family history of kidney stones also significantly increased the risk, with an odds ratio of 3.085 (Exp(B) = 3.085, 95% CI: 1.738-5.476, p < 0.001). Other variables, such as inflammatory bowel disease, diabetes, gout, chronic kidney disease, thyroid disorders, and the use of vitamin D, calcium pills, or diuretics, did not show significant associations with kidney stone development.

**Table 5:** Adjusted risk factors related to predictors of kidney stones (multivariate analysis)

Risk factor	B	Sig.	Exp(B)	95% CI
Smoking	0.575	0.033	1.776	1.047 – 3.015
Inflammatory bowel disease	0.099	0.882	1.104	0.300 – 4.062
Hypertension (HTN)	1.235	0.001	3.439	1.663 – 7.110
Diabetes	0.207	0.595	1.230	0.572 – 2.645
Gout	-0.372	0.619	0.689	0.159 – 2.981
Chronic kidney disease	0.579	0.407	1.784	0.454 – 7.009
Hyperthyroidism	0.777	0.259	2.175	0.563 – 8.398
Hypothyroidism	-0.279	0.705	0.756	0.179 – 3.204
Hyperparathyroidism	-0.702	0.421	0.496	0.090 – 2.740
Use of vitamin D pills	-0.536	0.234	0.585	0.242 – 1.414
Use of calcium pills	-0.709	0.191	0.492	0.170 – 1.423
Use of diuretics	-0.537	0.543	0.584	0.103 – 3.306
Family history of kidney stones	1.126	0.000	3.085	1.738 – 5.476
Constant	-0.424	0.005	0.655	

## Discussion

Kidney stones, or nephrolithiasis, involve crystal formation in the kidneys, primarily calcium stones (80%) composed of calcium oxalate or phosphate [11]. Factors such as urine supersaturation, pH levels, and diet contribute to their formation. Symptoms include flank pain, hematuria, urinary tract infections, and obstructive uropathy [12]. Previous literature indicates that calcium stones are the most common type, followed by uric acid, struvite, and cysteine stones [13]. Risk factors include gender, diet, climate, low fluid intake, and metabolic syndrome [14]. Kidney stones are a significant health concern in Saudi Arabia, with limited studies exploring their prevalence, incidence, and risk factors locally. This study aimed to evaluate the prevalence and associated risk factors of kidney stones among participants from the Jazan region, Saudi Arabia.

Notably, the prevalence of kidney stones in this study (55.4%) is significantly higher than global and regional prevalences. For example, a study conducted in Riyadh, Saudi Arabia, reports a prevalence of 9.4% [15], and worldwide prevalence estimates generally range between 1% and 15% depending on geographic and demographic variations [15]. The higher prevalence observed in our study may be attributed to environmental factors unique to the Jazan region, such as high temperatures leading to dehydration, dietary practices, and genetic predispositions.

Moreover, our findings reveal that males had significantly higher odds of developing kidney stones than females, with an odds ratio (OR) of 2.758 (p < .001). This aligns with numerous studies reporting a male predominance in kidney stone formation, attributed to differences in diet, hormonal influences, and higher urinary calcium excretion in males [16]. However, some studies suggest that the gender gap may be narrowing due to lifestyle and dietary changes and increasing prevalence in women [17].

Notably, age was another significant predictor, with each additional year increasing the odds of kidney stones by 49.3% (p = .003). Older age groups, particularly those above 50 years, had the highest prevalence. This is consistent with previous literature, where kidney stone risk increases with age, peaking in the fourth to sixth decades of life [18]. The underlying mechanism may involve age-related physiological changes, such as reduced renal function and changes in urinary composition.

Educational attainment was identified as a protective factor, with higher education reducing the odds of kidney stones by 44% (p < .001). This finding contrasts with previous studies suggesting that higher education correlates with better awareness of kidney stone prevention and healthier dietary habits [19]. Employment outside the health sector also increases the risk of kidney stones, possibly due to less access to health education and preventive care.

Interestingly, nationality and BMI did not significantly affect kidney stone prevalence in our study. This contrasts with findings from other research indicating a strong association between obesity and kidney stones [20]. The lack of significance in BMI could reflect the sample's specific characteristics or confounding factors unique to this population.

Among the risk factors analyzed, smoking was significantly associated with kidney stone prevalence, with smokers having 1.776 times the odds compared to non-smokers (p = .033). This aligns with findings that smoking increases oxidative stress, impairs renal function, and promotes stone formation [21].

Hypertension emerged as one of the strongest predictors, with individuals with hypertension showing 3.439 times higher odds of kidney stones (p = .001). The association between hypertension and kidney stones is well-documented, with shared pathophysiological mechanisms such as altered calcium metabolism and renal tubular dysfunction [22].

Family history was another significant predictor, with an odds ratio of 3.085 (p < .001). This finding supports previous studies emphasizing the hereditary component of kidney stone disease [23]. Genetic predispositions likely interact with environmental factors, increasing susceptibility in individuals with a family history [24].

Conversely, other conditions such as diabetes, gout, chronic kidney disease, and thyroid disorders did not show significant associations in our adjusted analysis. This contrasts with studies linking diabetes and gout to kidney stones, possibly due to differences in sample size or population characteristics [25, 26]. Additionally, the use of vitamin D and calcium supplements did not significantly influence kidney stone prevalence, suggesting that their effects might be dose-dependent, vary across populations, or not be fully understood [27].

This study has several strengths, including a robust sample size and a comprehensive analysis of sociodemographic and risk factors. However, some limitations must be acknowledged. The cross-sectional design limits causal inferences, and self-reported data on kidney stones and risk factors may be subject to recall bias. Furthermore, the absence of dietary data, such as fluid intake and oxalate consumption, restricts our understanding of specific environmental influences. Future studies should address these limitations by incorporating longitudinal designs and detailed dietary assessments.

## Conclusions

This study demonstrates a high prevalence of kidney stones among participants in the Jazan region, with significant sociodemographic and risk factors influencing their occurrence. Smoking, hypertension, and family history are key predictors, while education appears to be a protective factor. These findings underscore the importance of tailored interventions to reduce the burden of kidney stones and improve population health. Future research should explore the role of dietary and environmental factors to provide a more comprehensive understanding of kidney stone formation in this region.

## Figures and Tables

**Figure 1:**
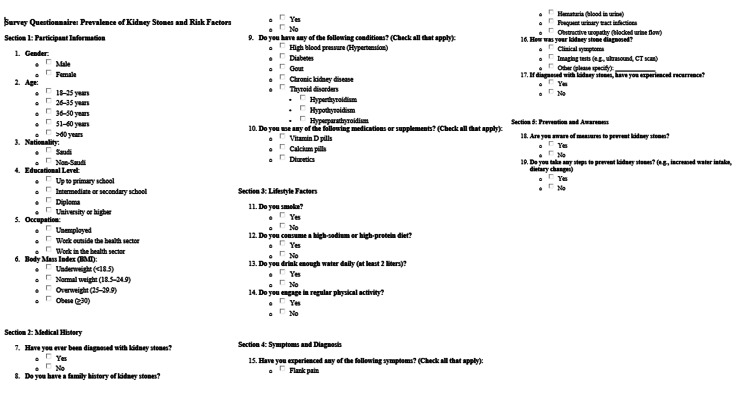
The questionnaire distributed to the study participants

## Appendices

Appendix A 
